# Effect of Magnetic Microparticles on Cultivated Human Corneal Endothelial Cells

**DOI:** 10.1167/tvst.12.2.14

**Published:** 2023-02-09

**Authors:** Joo-Hee Park, Kangmin Lee, Choul Yong Park

**Affiliations:** 1 Department of Ophthalmology, Dongguk University, Ilsan Hospital, Goyang, South Korea

**Keywords:** corneal transplantation, corneal endothelial cells, magnetic particle, nanoparticle, control

## Abstract

**Purpose:**

To investigate effects of magnetic microparticles on movement of magnet controlled human corneal endothelial cells (HCECs).

**Methods:**

Immortalized HCEC line (B4G12) and primary culture of HCECs were exposed to two commercially available magnetic micro- or nanoparticles, SiMAG (average size 100 nm) and fluidMAG (average size <1000 nm). Cell viability assays and reactive oxygen species production assays were performed. Cellular structural changes, intracellular distribution of microparticles, and expression levels of proteins related to cellular survival were analyzed. Ex vivo human corneas were exposed to microparticles to further evaluate their effects. Magnetic particle–laden HCECs were cultured under the influence of a neodymium magnet.

**Results:**

No significant decrease of viability was found in HCECs after exposure to both magnetic particles at concentrations up to 20 µg/mL for 48 hours. However, high concentrations (40 µg/mL and 80 µg/mL) of SiMAG and FluidMAG significantly decreased viability in immortalized HCECs, and only 80 µg/mL of SiMAG and FluidMAG decreased viability in primary HCECs after 48 hours of exposure. There was relative stability of viability at various concentrations of magnetic particles, despite a dose-dependent increase of reactive oxygen species, lactate dehydrogenase, and markers of apoptosis. Ex vivo human cornea study further revealed that exposure to 20 µg/mL of SiMAG and fluidMAG for 72 hours was tolerable. Endocytosed magnetic particles were mainly localized in the cytoplasm. The application of a magnetic field during cell culture successfully demonstrated that magnetic particle–loaded HCECs moved toward the magnet area and that the population density of HCECs was significantly increased.

**Conclusions:**

We verified short-term effects of SiMAG and fluidMAG on HCECs and their ability to control movement of HCECs by an external magnetic field.

**Translational Relevance:**

A technology of applying magnetic particles to a human corneal endothelial cell culture and controlling the movement of cells to a desired area using a magnetic field could be used to increase cell density during cell culture or improve the localization of corneal endothelial cells injected into the anterior chamber to the back of the cornea.

## Introduction

Human corneal endothelial cells (HCECs) are essential to maintain corneal transparency.^1^ HCEC density is the highest at birth and gradually decreases throughout life owing to a lack of proliferative capacity.^2^ Various pathologic conditions, including genetic and traumatic disease, can accelerate the loss of HCECs.^3^ When HCEC loss reaches a critical point, a very low density of HCECs can impair fluid pumping from the corneal stroma and results in corneal edema, leading to vision loss. If decompensation of HCECs occurs, the only treatment option is to use donor corneal tissue to replace them.^4^^–^^8^ Although still in experimental stages as potential types of therapy, HCECs can also be cultivated on biologic or polymeric carriers, such as decellularized stromal disc, collagen matrix, or hydrogel membrane, and can be transplanted as a lamellar graft.^5^^–^^7^

HCEC injection therapy is a novel technique.^9^^–^^12^ The most prominent advantage of injection therapy is that this technique eliminates the need for HCEC carriers such as a collagen sheet or amniotic membrane. HCECs directly injected to the anterior chamber can settle on the posterior corneal surface and are expected to restore fluid pumping action to dehydrate the corneal stroma. Results from a successful human trial are encouraging, with rapid restoration of vision and long-term maintenance.^11^ For the success of cell injection therapy, it is essential to allow as many cells as possible to settle on the posterior surface of the cornea to maximize the fluid pumping action of HCECs and to restore corneal transparency.

Improving culture efficiency and migration control of HCECs could further improve the success rate of cell injection therapy or tissue engineered HCEC constructs. Recently, there have been novel attempts to apply magnetic particles to HCECs and control cellular attachment on the corneal posterior surface.^9^^,^^10^^,^^13^ Several previous studies have evaluated the safety and efficacy of this technique by loading various types of magnetic particles into different animal-derived corneal endothelial cells, reflecting the great interest of many researchers in this field.^10^^,^^14^^,^^15^ Although the preliminary results of these new attempts are promising, thorough validation of safety and efficacy is essential before clinical application.

In this study, we used chemically synthesized commercial magnetic micro/nanoparticles and investigated their dose-dependent effect on HCECs. We used both cells from an established HCEC line (B4G12) and primary cultured HCECs obtained from fresh human donor corneas. Intracellular location of these magnetic particles and their possible dose-dependent toxicity were evaluated. In addition, we evaluated the possibility of endocytosed magnetic micro/nanoparticles to control HCECs by applying a neodymium magnet on the HCEC culture plate and verified that the cellular density of HCECs was significantly increased near the magnet area.

## Methods

All human corneas used in this research were provided by Eversight International (Seoul, South Korea). This research adhered to the tenets of the Declaration of Helsinki with the approval of the Institutional Review Board of Dongguk University, Ilsan Hospital, Goyang, South Korea. Informed consent regarding the possible research use of the corneas was obtained from donors after they agreed to donate tissues.

### Magnetic Micro/Nanoparticles

Two commercially available magnetic particles, SiMAG (SiMAG-silanol, catalog no 1101-5) and fluidMAG (fluidMAG-D, catalog no 4101-1) (Chemicell GmbH, Berlin, Germany), were purchased. SiMAG is a silanol bead that has an unmodified silica surface with terminal negatively charged silanol groups and iron oxide cores (5–20 nm). FluidMAG is a ferrofluid consisting of an aqueous dispersion of magnetic iron oxide cores (5–20 nm) covered by a hydrophilic polymer. In this study, the average size of fluidMAG was approximately 100 nm and the average size of SiMAG was less than 1000 nm according to the company-provided data sheet.

### HCEC Culture

An established HCEC line, B4G12 (Cat no. CSC-C3457-CRA), was purchased from Creative Bioarray (Shirley, NY). These cells were cultured using the medium recommended by the company, which contained human endothelial serum-free medium (Creative Bioarray, Cat no. CM-345L7) and 10 ng/mL of fibroblast growth factor-2 (Creative Bioarray, Cat no. CSC-CTK0134). The culture medium was changed every three days. Cells were passaged using 0.25% Trypsin-EDTA (Gibco BRL, Carlsbad, CA).

Primary HCEC culture was performed using both corneas from a 51-year-old female donor according to previously described peel-and-digest modified methods.^16^^–^^18^ Endothelial density of donor corneas were 3125 and 3049/mm^2^, respectively. The death to preservation time was 9 hours and the cell culture was started at day 8 after the death of the donor. Descemet's membranes were carefully peeled off together with endothelial cells from the corneal stroma and were briefly rinsed using 1× antibiotic-antimycotic solution (Gibco). Then they were digested in 1 mg/mL collagenase type I (Sigma-Aldrich, St. Louis, MO) for 4 hours at 37°C in a 5% CO_2_ incubator. After washing with Dulbecco's phosphate-buffered saline (DPBS), HCECs were dissociated with 0.05% Trypsin-EDTA (Gibco) for 5 minutes using pipetting. Fully isolated HCECs were centrifuged at 500×*g* for 5 minutes, and the cell pellet was cultured and maintained in FNC coating mix (AssayCell Technologies, Cat#ACT1-0407, Avenel, NJ) coated plates with endothelial cell growth medium-2 (EGM-2; Lonza, Cat# CC-3162, Walkersville, MD) supplemented with 10 µM Rho-associated kinase inhibitor. After 48 hours of attachment, the growth media, except Rho-associated kinase inhibitor, were changed every 2 to 3 days until HCECs reached confluency at 10 to 14 days. Cells were subcultured after reaching 80% to 90% confluence by treating with 0.25% trypsin-EDTA and seeded at a split ratio of 1:3. Cell cultures and passages were carried out at 37°C in a 5% CO_2_ incubator. In this study, we used primary cultured HCECs at passage four.

### Cell Viability Assay

The viability of HCECs was determined using a commercial cell viability reagent, PrestoBlue (Invitrogen, Eugene, OR). HCECs were cultured and seeded into a 96-well plate at a density of 2 × 10^4^ cells/well. After cell attachment, SiMAG and fluidMAG were used to treat cells at concentrations of 0.00, 1.25, 2.50, 5.00, 10.00, 20.00, 40.00, and 80.00 µg/mL for 24 to 72 hours. After appropriate incubation, 10 µL of PrestoBlue reagent was added to each culture well. After 1 hour of incubation with the reagent at 37°C, absorbance at 570 nm was measured with absorbance at 600 nm as a reference.

### Lactate Dehydrogenase (LDH) Assay

LDH in HCECs after exposure to magnetic particles was measured using an LDH cytotoxicity detection kit (Takara Bio Inc., Shiga, Japan). Experiments were performed according to the manufacturer's protocol. HCECs were cultured and incubated at 2 × 10^4^ cells/well in a 96-well plate. After cell attachment, SiMAG and fluidMAG were applied to cells at concentrations of 0.00, 1.25, 2.50, 5.00, 10.00, 20.00, 40.00, and 80.00 µg/mL for 24 to 72 hours. Wells to which nanoparticles were not added and those to which 1% Triton X-100 was added were used as negative and positive controls, respectively. After cell incubation, the cell-free supernatant was transferred to a new 96-well plate. Wells were incubated with the reaction mixture at room temperature (RT) for 20 minutes. Absorbance was measured at a wavelength of 490 nm.

### Measurement of Reactive Oxygen Species (ROS)

Intracellular ROS were measured using a 2′,7′-dichlorofluorescin diacetate (DCFDA)/H2DCFDA-Cellular ROS Assay Kit (Cat. No. ab113851: Abcam, Cambridge, UK). HCECs were treated with SiMAG and fluidMAG at various concentrations (0.00, 1.25, 2.50, 5.00, 10.00, 20.00, 40.00, and 80.00 µg/mL) for 24, 48, and 72 hours. After treatment, the media were removed and cells were washed using 100 µL/well of 1× buffer according to the manufacturer's protocol. Cells were stained at 37°C in the dark for 45 minutes by adding 100 µL/well of a diluted 20 µM solution of DCFDA. They were then removed from the solution and 100 µL/well of 1× buffer was added. Finally, the fluorescence was measured immediately at 485 nm excitation/535 nm emission in an end point mode.

### Electron Microscopy Analysis

The distribution of SiMAG or fluidMAG in HCECs was investigated using a transmission electron microscope (TEM), as previously described.^19^ HCECs were treated with SiMAG or fluidMAG (20 µg/mL) for 24 hours. Cells were fixed overnight with 2.5% glutaraldehyde (Sigma-Aldrich) and 3.7% paraformaldehyde (Sigma-Aldrich) in 0.1 M phosphate buffer (pH 7.6). After washing with 0.1 M phosphate buffer, HCECs were fixed with 1% osmium tetroxide (OsO_4_) in the same buffer for 1 hour. Cells were then dehydrated with a graded ethanol series (Merck KGaA, Darmstadt, Germany) before embedding in an epoxy embedding medium (Sigma-Aldrich). Polymerization was then performed at 60°C for 3 days. Ultrathin sections (60–70 nm) of samples were obtained with an ultramicrotome (Leica Ultracut UCT, Leica, Germany). Sections were collected on a grid (200 mesh) and examined using TEM (JEM-1010; JEOL, Tokyo, Japan) at 60 kV. Images were recorded with a charge-coupled device camera (SC1000; Gatan, Warrendale, PA). The length of the electron micrograph was measured with the Gatan Microscopy Suite software (Gatan, Warrendale, PA). Normal control for TEM was incubated in a complete nanoparticle-free medium for 24 hours.

### Western Blot Analysis

Western blot analysis was performed in accordance with a previously reported method.^19^ All magnetic particle-treated cells were lysed with an ice-cold radioimmunoprecipitation assay buffer (50 mM Tris-HCl [pH 8.0], 150 mM NaCl, 1% NP-40, 0.5% deoxycholate, and 0.1% sodium dodecyl sulfate) for 30 minutes. The debris was removed by centrifugation at 16,000×*g* for 1 minute. Equal amounts (20 µg) of total cell protein were separated by sodium dodecyl sulfate–polyacrylamide gel electrophoresis and transferred to a polyvinylidene difluoride membrane (Millipore Corporation, Billerica, MA). After blocking with 3% bovine serum albumin (Sigma-Aldrich) in Tris-buffered saline (10 mM Tris, pH 8.0, 150 mM NaCl) with 0.1% Tween 20 at RT for 1 hour, membranes were incubated overnight at 4°C with the following primary antibodies: rabbit anti–Bcl-2 associated X protein (BAX) (1:1000; Cat. No. 2772; Cell Signaling, Beverly, MA), rabbit anti–B-cell lymphoma (Bcl)-/xL (1:1000; Cat. No. 2764; Cell Signaling), mouse antinuclear factor erythroid-2-related factor 2 (Nrf2) (1:1000; Cat. No. MAB3925; R&D system, Minneapolis, MN), rabbit antinicotinamide adenine dinucleotide phosphate quinone oxidoreductase 1 (NQO1) (1:1000; Cat. No. PA521290; Thermo Fisher Scientific, Waltham, MA), rabbit antiphosphorylated-p44/42 mitogen-activated protein kinase (extracellular signal regulated kinase [ERK] 1/2) (1:1000; Cat. No. 4370; Cell Signaling), rabbit anti-p44/42 mitogen-activated protein kinase (ERK 1/2) (1:1000; Cat. No. 4695; Cell Signaling), and mouse anti–β-actin (1:50,000; Cat. No. A5441; Sigma-Aldrich). Membranes were then incubated with horseradish peroxidase-conjugated secondary antibodies at RT for 1 hour. Blots were developed with an enhanced chemiluminescence kit (Cat. No. RPN2232; GE Healthcare, Buckinghamshire, UK) and visualized with a Fujifilm LAS-3000 image reader (Fujifilm, Tokyo, Japan). Densitometric analysis was performed with a Multi Gauge V3.0 software (Fujifilm Life Science, Tokyo, Japan). Each experiment was performed in triplicate at a minimum.

### Immunocytochemistry

As previously reported,^19^ HCECs were seeded at a density of 4 × 10^4^ cells/mL, grown on 4-well Lab-Tek chamber slides (Nalgene Nunc International, Penfield, NY), and treated with 20 µg/mL of SiMAG or fluidMAG for 24 hours. HCECs were fixed with 3.7% paraformaldehyde at RT for 10 minutes. Permeabilization with 0.1% Triton X-100 was performed for 5 minutes at RT. After washing with DPBS, nonspecific antigen sites were blocked with 1% bovine serum albumin in DPBS at RT for 30 minutes. Chamber slides were incubated with rabbit polyclonal anti–ZO-1 (1:500; Cat. No. 13663; Cell Signaling) at 4°C overnight. Next, slides were washed with DPBS and incubated with Alexa 488-conjugated donkey anti-rabbit antibody (1:1000; Cat. No. A21206; Invitrogen) at RT for 1 hour. Tetramethylrhodamine isothiocyanate (TRITC)-conjugated phalloidin (1 µg/mL; Sigma-Aldrich) was used to stain F-actin. Cell nuclei were counterstained using 4ʹ,6-diamidino-2ʹ-phenylindole (DAPI, Cat. No. 10236276001; Roche, Mannheim, Germany). Slides were viewed using a fluorescence microscope (Olympus BX53F, Tokyo, Japan).

### Ex Vivo HCEC Toxicity Assay

Six donor corneas were used for the ex vivo experiment. Two corneas were from a 19-year-old female donor. Another two corneas were from a 31-year-old female donor. One cornea was from a 22-year-old male donor and one cornea was from a 35-year-old male donor. These corneas were harvested on the date of donor death and used for the experiment at 8 to 12 days after death. Four round corneas (3 mm in diameter) were harvested using a corneal punch from each human corneal donor button. Each segment was stained with 0.005% trypan blue mixed with minimum essential medium (MEM) for 5 minutes. Trypan blue staining is a long-standing and widely used method to identify dead cells. Corneal endothelial cell viability was assessed by examining blue-stained area under an inverted-phase contrast microscope. After baseline viability assessment, corneal segments were incubated in culture media containing various concentrations of microparticles (SiMAG or fluidMAG: 0, 20, 40, and 80 µg/mL) at 37°C in a 5% CO_2_ and 95% air-humidified atmosphere for 72 hours. The tissue culture medium was serum-free MEM containing L-glutamine (2 mM), NaHCO_3_ (20 g/L), penicillin (100 IE/mL), and streptomycin (0.1 mg/mL). After 72 hours of incubation, corneal segments were stained again with 0.005% trypan blue mixed with MEM for 5 minutes. Any increase in blue-stained area from baseline was used as an indicator of corneal endothelial cell toxicity. These increases were compared among groups. For more visualization of the trypan blue stained area, 0.01% trypan blue mixed with MEM for 5 minutes was applied to cornea exposed to SiMAG or fluidMAG at 40 or 80 µg/mL. After trypan blue evaluation, corneal segments were fixed with 10% formalin and incubated with TRITC-conjugated phalloidin (1 µg/mL; Sigma-Aldrich). To detect apoptosis in HCECs, a terminal deoxynucleotidyl transferase dUTP nick-end labeling assay was performed using an APO-BrdU dUTP nick-end labeling assay kit (Cat. No. A23210: Invitrogen) in accordance with the manufacturer's protocol. Tissues were rinsed with PBS three times (5 minutes per rinse). Whole corneas were mounted with the endothelial-side down on slides and stained with DAPI (Roche). These slides were then examined with a fluorescence microscope.

### Cell Migration Assay

HCECs were seeded at a density of 3 × 10^4^ cells/mL and grown on 4-well Lab-Tek chamber slides (Nalgene Nunc International). They were then treated with 20 or 40 µg/mL of SiMAG or fluidMAG for 24 hours. Residual SiMAG and fluidMAG were removed using DPBS (Supplementary Fig. S2). Customized neodymium magnets with a diameter of 10 mm (JL magnet, Seoul, Korea) were then attached under the chamber slide for 7 days. Culture media were changed every 3 days. The level of migration was observed using immunocytochemistry on day 7. Cultured HCECs were fixed with 3.7% paraformaldehyde at RT for 10 minutes. Permeabilization was performed with 0.1% Triton X-100 for 5 minutes at RT. Immortalized HCECs (B4G12) were incubated with TRITC-conjugated phalloidin (1 µg/mL; Sigma-Aldrich). Primary cultured HCECs were incubated with Phalloidin Atto 488 (1:500; Cat. NO. 49409; Sigma-Aldrich) for F-actin staining. Nuclei were stained using DAPI. Slides were then viewed using a fluorescence microscope.

### Statistical Analyses

All data are presented as mean ± standard error. Statistical significance was determined by analysis of variance and Dunnett's multiple comparison test. *P* values of less than 0.05 were regarded as significant. All statistical analyses were performed using GraphPad Prism Ver. 9.3.1 (GraphPad Software Inc., La Jolla, CA).

## Results

### Intracellular Distribution of Magnetic Particles

**Figure 1.**
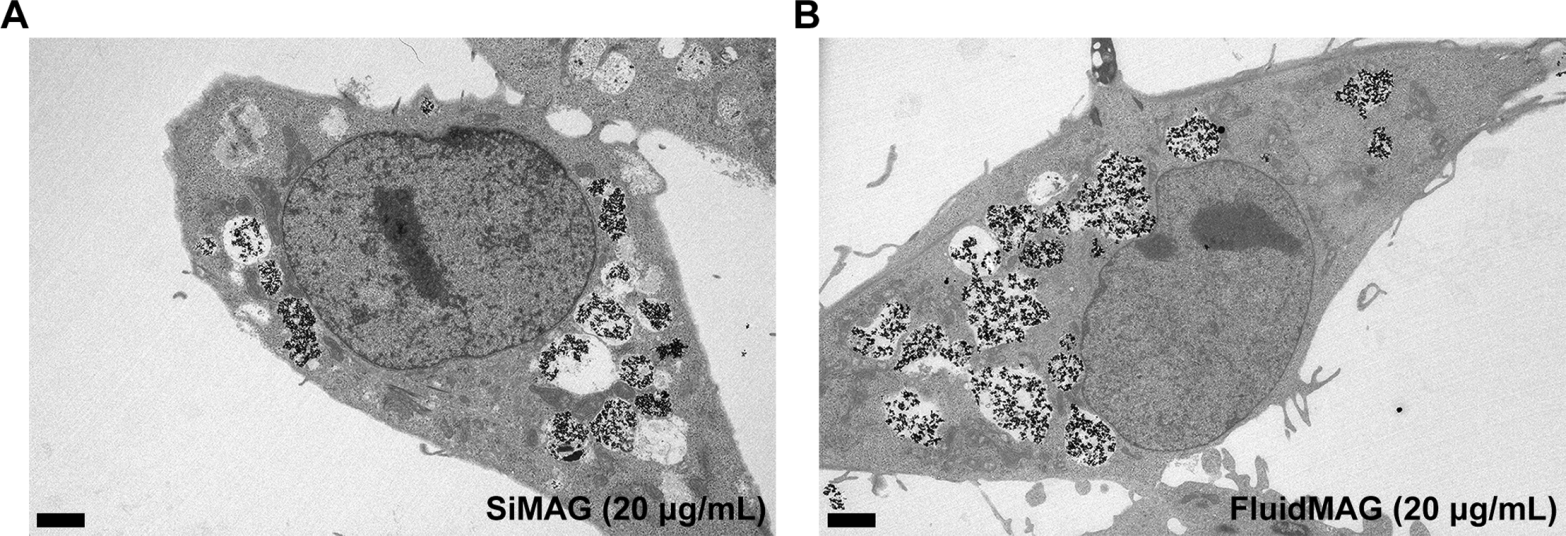
Human corneal endothelial cells (B4G12 cells) treated with SiMAG (**A**) and fluidMAG (**B**) for 24 hours. Intracellular distribution of SiMAG and fluidMAG was demonstrated by transmission electron microscopy. Particles clustered and remained in the cytoplasm. No definite nuclear entry was observed. Structures of intracellular organelles were well maintained. Scale bar, 2 µm.

Magnetic particles were observed mainly in cytoplasmic vesicles of HCECs. The silica structure of SiMAG and hydrophilic polymer coat of fluidMAG were unrecognizable in TEM images. Only 5- to 20-nm-sized iron oxide nanoparticles could be detected. Cellular ultrastructures such as mitochondria and nucleus were well-maintained, even with abundant cytoplasmic magnetic nanoparticles (Fig. 1). No nuclear entry of particles was observed. The zeta potential provides useful information for estimating dispersion of particles in a solution state. A zeta potential analysis was performed by requesting Korea polymer testing and research institute, Ltd. (www.polymer.co.kr/eng/sub). The zeta potential of SiMAG was measured as −185.75 mV in distilled water and −37.35 mV in culture media. The zeta potential of fluidMAG was measured as −80.75 mV in distilled water and −18.65 mV in culture media.

### Cell Viability, ROS, and LDH Assay

**Figure 2.**
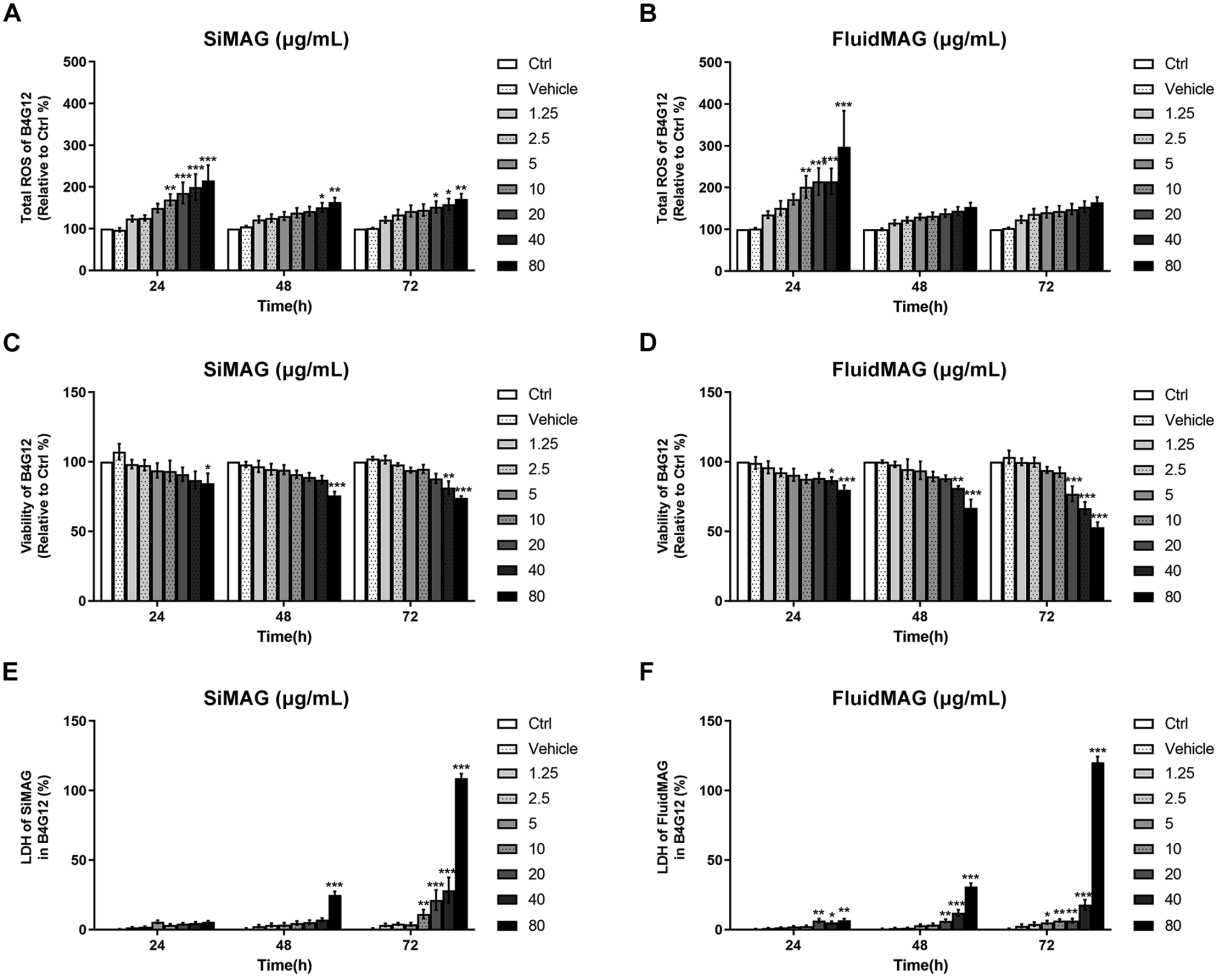
ROS generation, cell viability, and LDH release from HCECs (B4G12 cells) measured after exposure to various concentrations of SiMAG and fluidMAG. (**A** and **B**) Both magnetic particles significantly increased ROS dose-dependently. The increase of ROS was more prominent after 24 hours of culture. Longer cultivation for 48 hours or 72 hours also dose-dependently increased ROS, although the overall response was slightly dampened. (**C** and **D**) Cell viability decreased after exposure to higher concentrations of SiMAG and fluidMAG. Exposure to 80 µg/mL of SiMAG and fluidMAG for 72 hours resulted in about 20% and 50% decreases in cell viability, respectively. (**E** and **F**) LDH release verified increased cellular death after exposure to higher concentrations of SiMAG and fluidMAG. The longer the exposure, the higher the cell toxicity. Ctrl, negative control with no addition of magnetic particles. Triplicates of each treatment group were used in each independent experiment. Values are presented as mean ± standard error of the mean from three independent experiments. *P < 0.05, **P < 0.01, ***P < 0.001.

**Figure 3.**
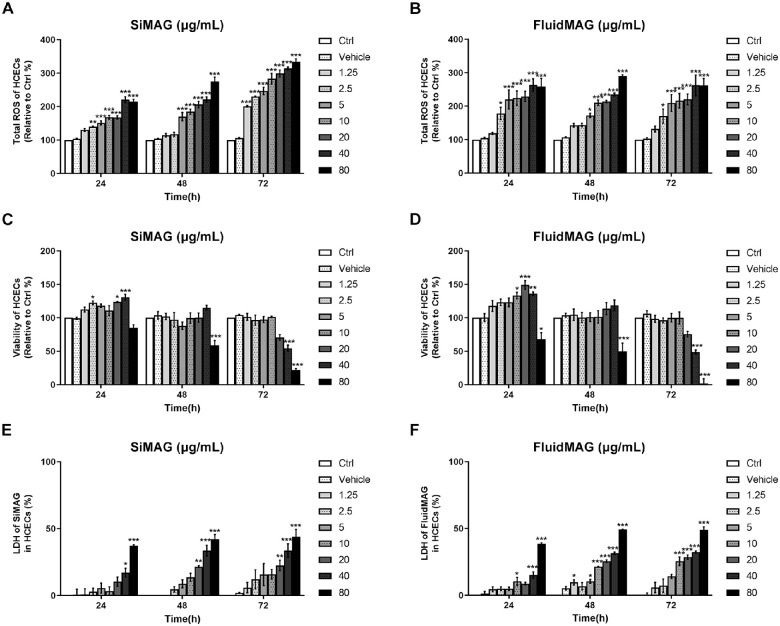
ROS generation, cell viability change, and LDH release from primary cultured HCECs after exposure to various concentrations of SiMAG and fluidMAG. (**A** and **B**) Both magnetic particles increased ROS dose-dependently. Exposure to SiMAG showed a tendency to increase ROS at higher concentrations and after longer cultivation. However, the ROS increase after exposure to fluidMAG was mainly dose dependent, not time dependent. (**C** and **D**) Cell viability decreased significantly after exposure to higher concentrations of SiMAG and fluidMAG. Exposure to 80 µg/mL of SiMAG and fluidMAG for 72 hours resulted in approximately 80% and 100% decreases in cell viability, respectively. (**E** and **F**) LDH release verified increased cellular death after exposure to higher concentrations of SiMAG and fluidMAG. Ctrl, negative control with no addition of magnetic particles. Triplicates of each treatment group were used in each independent experiment. Values are presented as mean ± standard error of the mean from three independent experiments. *P < 0.05, **P < 0.01, ***P < 0.001.

Both SiMAG and fluidMAG showed dose-dependent cytotoxicities to HCECs. Toxic effects of magnetic particles were more prominent in the primary culture of HCECs than in immortalized HCECs (B4G24 cells). Both magnetic particles dose-dependently increased cellular ROS. Exposure to SiMAG (80 µg/mL) increased ROS to 215% (*P* < 0.001), 163% (*P* < 0.01), and 171% (*P* < 0.05) at 24 hours, 48 hours, and 72 hours after incubation, respectively. Exposure to fluidMAG (80 µg/mL) increased ROS to 297% (*P* < 0.001), 153% (*P* < 0.01), and 164% (*P* < 0.05) at 24 hours, 48 hours, and 72 hours after incubation, respectively (Fig. 2). Interestingly, magnetic particle-induced ROS increase was more prominent after a short exposure time (24 hours) in immortalized HCECs. Such an effect was slightly decreased when the exposure time was prolonged to 48 hours or 72 hours. However, this dampening effect of ROS by prolonged culture duration was not observed in primary cultured HCECs. Consequently, 72 hours of exposure to higher concentrations (>40 µg/mL) of magnetic particles resulted in drastic toxicity to both immortalized and primary cultures of HCECs (Figures 2 and 3). We found that immortalized HCECs maintained stable viability for 48 hours when SiMAG and fluidMAG concentrations were up to 20 µg/mL. We also found that the viability of primary culture of HCECs was stable for 48 hours when cells were exposed to SiMAG and fluidMAG at concentrations of up to 40 µg/mL. Extending the exposure duration to 72 hours resulted in a more prominent decrease of cell viability. Exposure of immortalized HCECs to 40 µg/mL of SiMAG for 72 hours resulted in 81% cell viability (*P* < 0.01). Exposure of immortalized HCECs to 80 µg/mL of SiMAG for 48 hours and 72 hours resulted in cell viabilities of 75% (*P* < 0.001) and 74% (*P* < 0.001), respectively. Exposure of primary cultured HCECs to 40 µg/mL of SiMAG for 72 hours resulted in 54% cell viability (*P* < 0.001). Exposure of primary cultured HCECs to 80 µg/mL of SiMAG for 48 hours and 72 hours resulted in cell viabilities of 58% (*P* < 0.001) and 22% (*P* < 0.001), respectively. Exposure of immortalized HCECs to 40 µg/mL of fluidMAG for 48 hours and 72 hours resulted in cell viabilities of 81% (*P* < 0.01) and 61% (*P* < 0.001), respectively. Exposure of immortalized HCECs to 80 µg/mL of fluidMAG for 24 hours, 48 hours, and 72 hours resulted in cell viabilities of 80% (*P* < 0.001), 66% (*P* < 0.001), and 52% (*P* < 0.001), respectively. Exposure of primary cultured HCECs to 40 µg/mL of fluidMAG for 72 hours resulted in a cell viability of 48% (*P* < 0.001). Exposure of primary cultured HCECs to 80 µg/mL of fluidMAG for 24 hours, 48 hours, and 72 hours resulted in cell viabilities of 68% (*P* < 0.05), 49% (*P* < 0.001), and 2% (*P* < 0.001), respectively. However, the viabilities of both immortalized and primary culture of HCECs were well-maintained after they were exposed to SiMAG or fluidMAG at concentrations up to 10 µg/mL for 72 hours. Exposure to 20 µg/mL of SiMAG or fluidMAG for 72 hours resulted in only a 25% loss of viability. LDH also increased at higher concentrations of magnetic particles with longer exposure time.

### Cytoskeletal Maintenance After Exposure to SiMAG and FluidMAG

**Figure 4.**
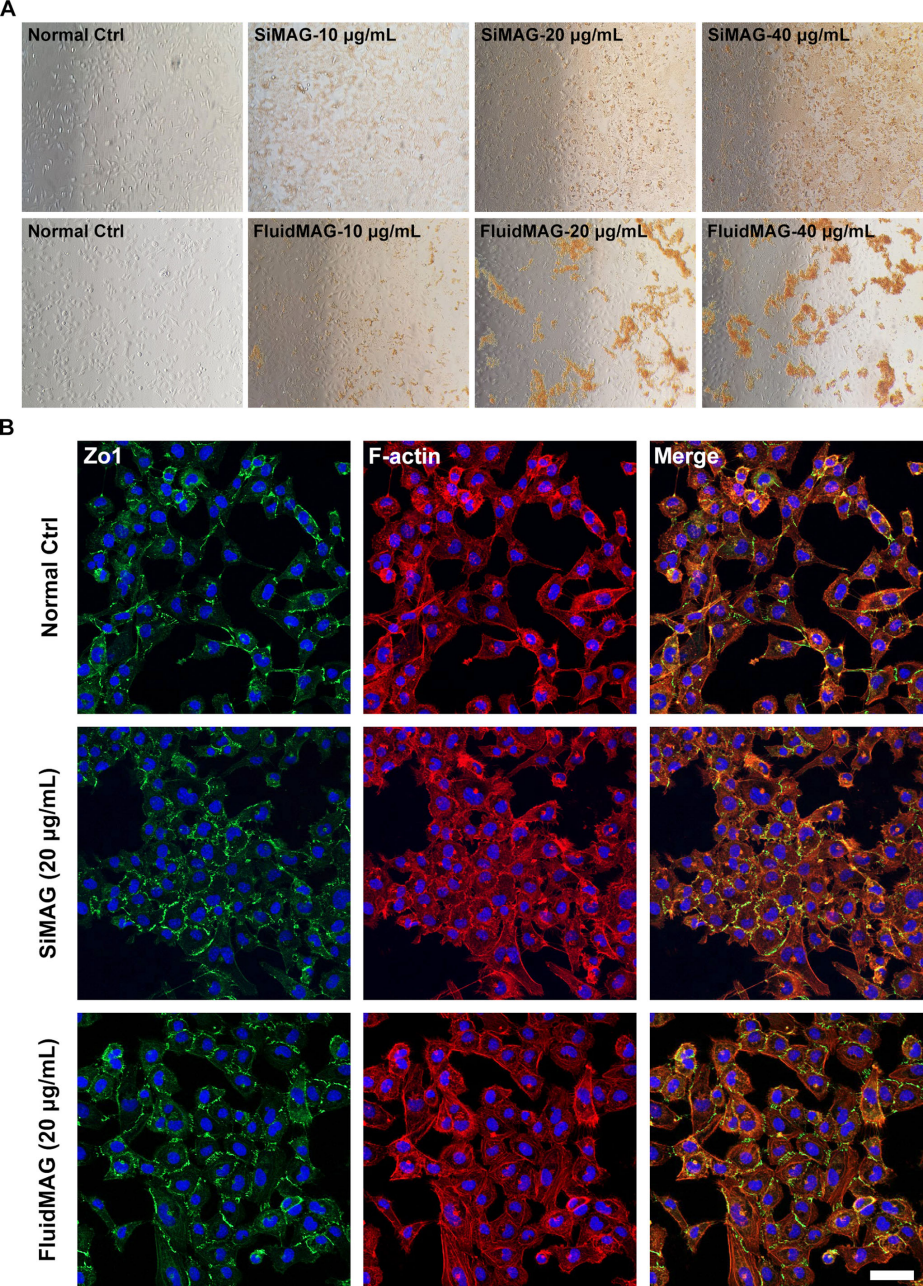
Expression levels of cytoskeleton (F-actin) and cell adhesion molecules (ZO-1) investigated by immunocytochemistry. Immortalized HCECs (B4G12 cells) were exposed to 10, 20, and 40 µg/mL SiMAG and fluidMAG for 24 hours. (**A**) A phase contrast microscope revealing a well-maintained cell structure. Brown pigment represents the color of magnetic microparticles. (**B**) Expression levels of ZO-1 and F-actin showed no significant difference when compared with the control (no exposure to any magnetic microparticles). Scale bar, 50 µm.

Changes in cytoskeleton (F-actin) and cell adhesion molecule (ZO-1) expression of immortalized HCECs (B4G24 cells) were evaluated after exposure to 20 µg/mL of SiMAG or fluidMAG. This concentration was selected because we observed a greater than 80% cell viability after 72 hours of exposure to SiMAG or fluidMAG. After exposure to 20 µg/mL of microparticles for 24 hours, the morphology of immortalized HCECs (B4G24 cells) was well-maintained and normal expression of F-actin and ZO-1 was verified (Fig. 4).

### Cellular Survival Signal Analysis After Exposure to SiMAG and FluidMAG

**Figure 5.**
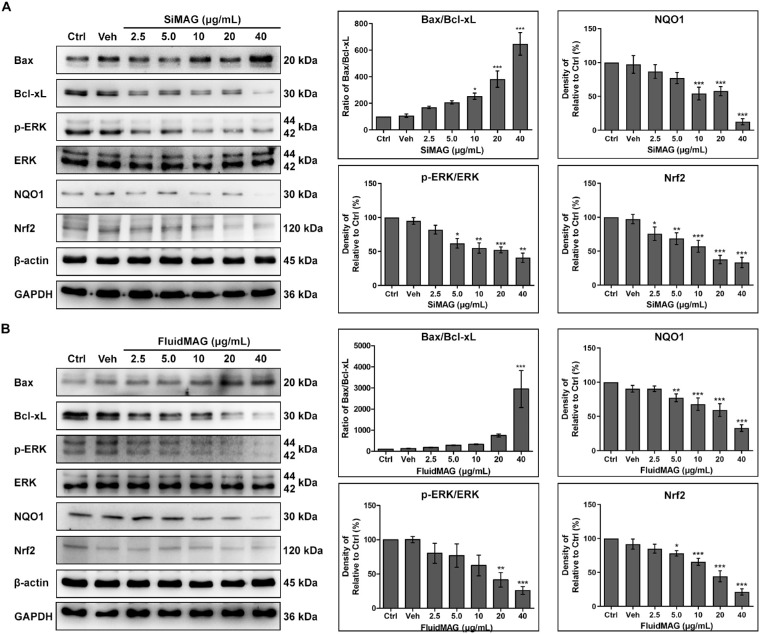
Exposure to magnetic particles (SiMAG [**A**] and fluidMAG [**B**]) results in a dose-dependent decrease in cellular survival signals and significant depletion of cellular antioxidant mechanisms in immortalized HCECs (B4G12 cells). After 24 hours of exposure to increasing concentrations of magnetic microparticles, cellular survival signals such as ERK phosphorylation (p-ERK) were decreased dose-dependently with increasing ratio (Bax/Bcl-xL) of proapoptotic (Bax) and antiapoptotic (Bcl-xL) protein expression. These toxicities were related to exhaustion of the antioxidant mechanism demonstrated by significant decreases of NQO1 and Nrf2 proteins. Note that only bands at adequate molecular weights are shown here. Full-length gel and blots are included in supplementary information. Ctrl, negative control with no addition of magnetic particles. Triplicates of each treatment group were used in each independent experiment. Values are presented as mean ± standard error of the mean from three independent experiments. *P < 0.05, **P < 0.01, ***P < 0.001.

Exposure of immortalized HCECs (B4G24 cells) to SiMAG or fluidMAG revealed a dose-dependent increase in Bax expression (proapoptotic) coupled with a decrease in Bcl-xL expression (antiapoptotic), resulting in an increase in the Bax/Bcl-xL ratio (Fig. 5). ERK phosphorylation was also decreased after cells were exposed to higher concentrations of SiMAG and fluidMAG. SiMAG induced a significant increase of Bax/Bcl-xL ratio from 10 µg/mL concentration. A decrease of ERK phosphorylation was observed from the 5 µg/mL concentration after 24 hours of exposure. The increase of Bax/Bcl-xL ratio was detected only at the 40 µg/mL concentration and the decrease of ERK phosphorylation was observed from the 20 µg/mL concentration after 24 hours of exposure to fluidMAG. Exposure to 40 µg/mL of SiMAG for 24 hours increased the Bax/Bcl-xL ratio to 647% (*P* < 0.001) and decreased p-ERK/ERK to 40.7% (*P* < 0.01), whereas exposure to 40 µg/mL of fluidMAG for 24 hours increased the Bax/Bcl-xL ratio to 2946.0% (*P* < 0.001) and decreased p-ERK/ERK to 25.9% (*P* < 0.01). Two cellular antioxidant components of NQO1 and Nrf2 decreased with higher concentrations of SiMAG and fluidMAG, indicating an excess consumption of these antioxidants. After 24 hours of exposure to 40 µg/mL of SiMAG, NQO1 and Nrf2 decreased to 12.5% (*P* < 0.001) and 33.4% (*P* < 0.001), respectively. After 24 hours of exposure to 40 µg/mL of fluidMAG, NQO1 and Nrf2 decreased to 33.0% (*P* < 0.001) and 21.3% (*P* < 0.001).

### Ex Vivo HCEC Toxicity Assay

**Figure 6.**
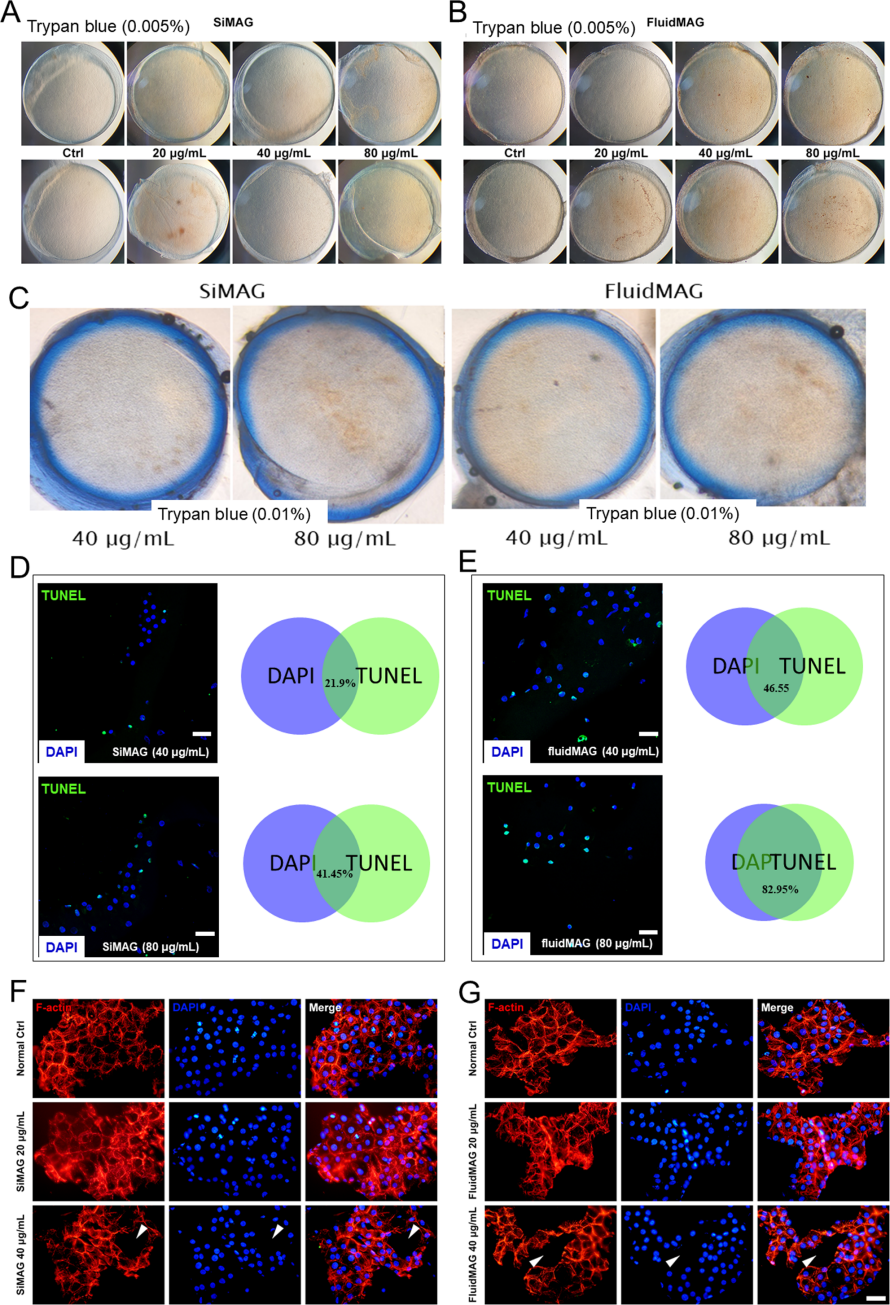
Effects of SiMAG and fluidMAG on ex vivo human corneas. (**A** and **B**) Trypan blue staining (0.005%) revealing a patchy area of HCEC staining after exposure to higher (40 and 80 µg/mL) concentrations of SiMAG and fluidMAG. **C**: More prominent patchy blue staining area with 0.01% trypan blue staining. (**D** and **E**) dUTP nick-end labeling staining revealing a significant proportion of HCECs under apoptosis after exposure to 40 or 80 µg/mL of SiMAG or fluidMAG. (**F** and **G**) F-actin and DAPI staining of corneas in panels A and B demonstrating an area void of HCECs (arrows). Scale bar, 50 µm.

The toxicities of SiMAG and fluidMAG were evaluated using ex vivo human corneas. Trypan blue normally stains damaged HCECs. This technique is useful for evaluating the quality of donor corneas before transplantation.^20^ After 24 hours of exposure to 20, 40, and 80 µg/mL of SiMAG or fluidMAG, ex vivo corneas were observed under a phase contrast microscope to determine damage to HCECs stained with trypan blue. Some patchy staining of trypan blue was observed after exposure to 40 or 80 µg/mL of SiMAG or fluidMAG (Figures 6A and 6B). Immunostaining with F-actin and DAPI revealed an area void of cells after exposure to 40 µg/mL of SiMAG or fluidMAG (Figures 6C and 6D).

### Control of Cell Culture Density by Magnetic Particle Application

Loading with magnetic particles (SiMAG or fluidMAG) significantly increased HCEC cell density near the neodymium magnet area. Exposure concentrations (20 µg/mL for immortalized HCECs and 40 µg/mL for primary culture of HCEC) for migration assay were determined according to the maximum safe exposure dose demonstrated in Figures 2 and 3.

**Figure 7.**
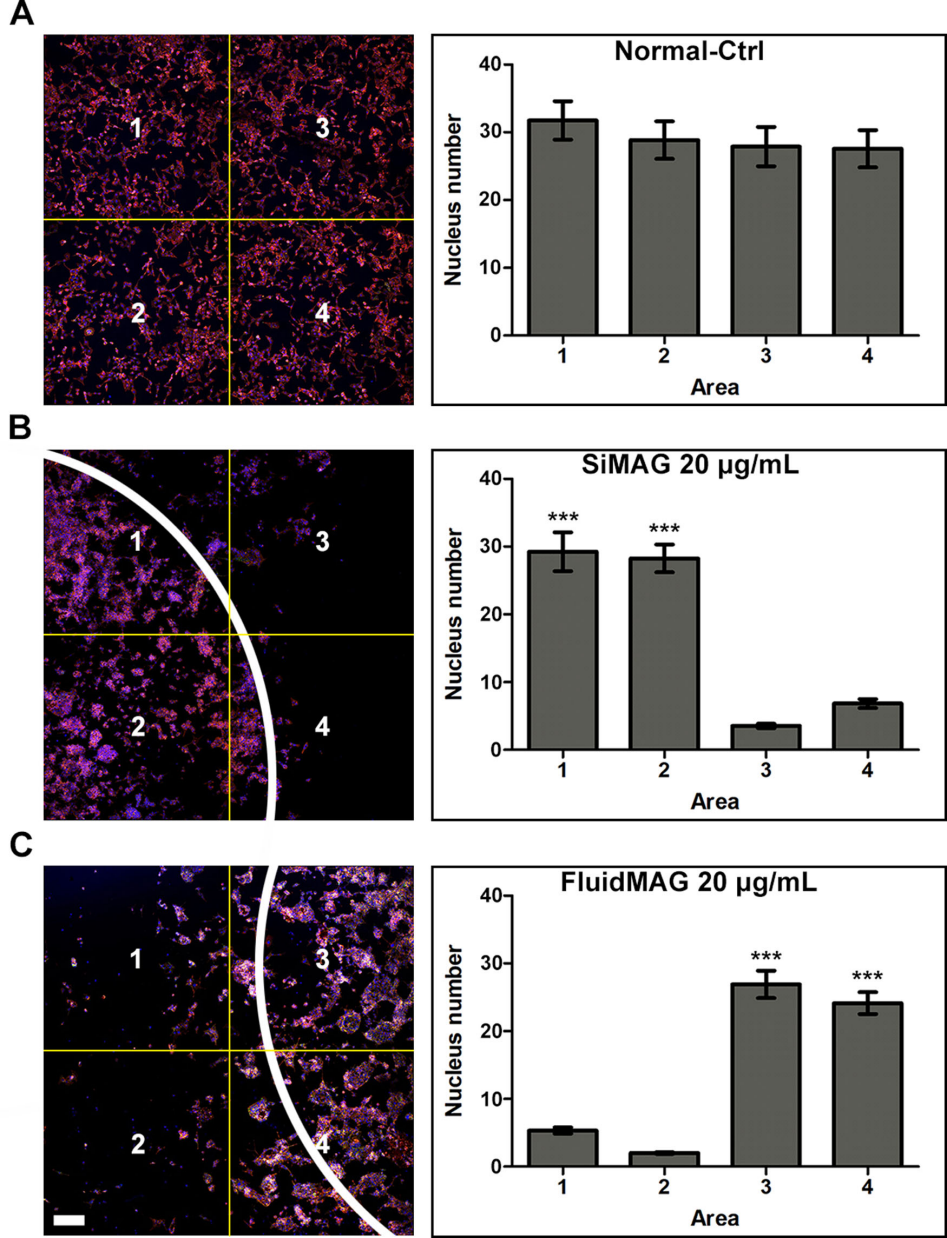
Control of cell growth density by application of magnetic particles. Magnetic particle-loaded immortalized HCECs (B4G12 cells) were suspended and cultured in the plate under which the round neodymium magnet was placed. Loading HCECs with 20 µg/mL of SiMAG or fluidMAG efficiently increased cell population near the magnet area. This suggests an effect of a magnetic field in attracting HCECs and controlling cell density. (**A**) Negative control with no loading of magnetic particles. Areas 1 to 4 show similar cell population. (**B**) HCECs loaded with 20 µg/mL of SiMAG. An increase of cell population was observed in areas 1 and 2. *P* values in (**B**) were calculated compared with the lowest populated area (area 3). (**C**) HCECs loaded with 20 µg/mL of fluidMAG. An increase of cell population was observed in areas 3 and 4. *P* values in (**C**) were calculated compared with the lowest populated area (area 2). The white arc in (**B** and **C**) indicates the margin of the round neodymium magnet. HCECs in (**A**–**C**) were stained with F-actin (red) and DAPI (blue). Triplicates of each treatment group were performed. Representative figures are presented. ***P < 0.001.

**Figure 8.**
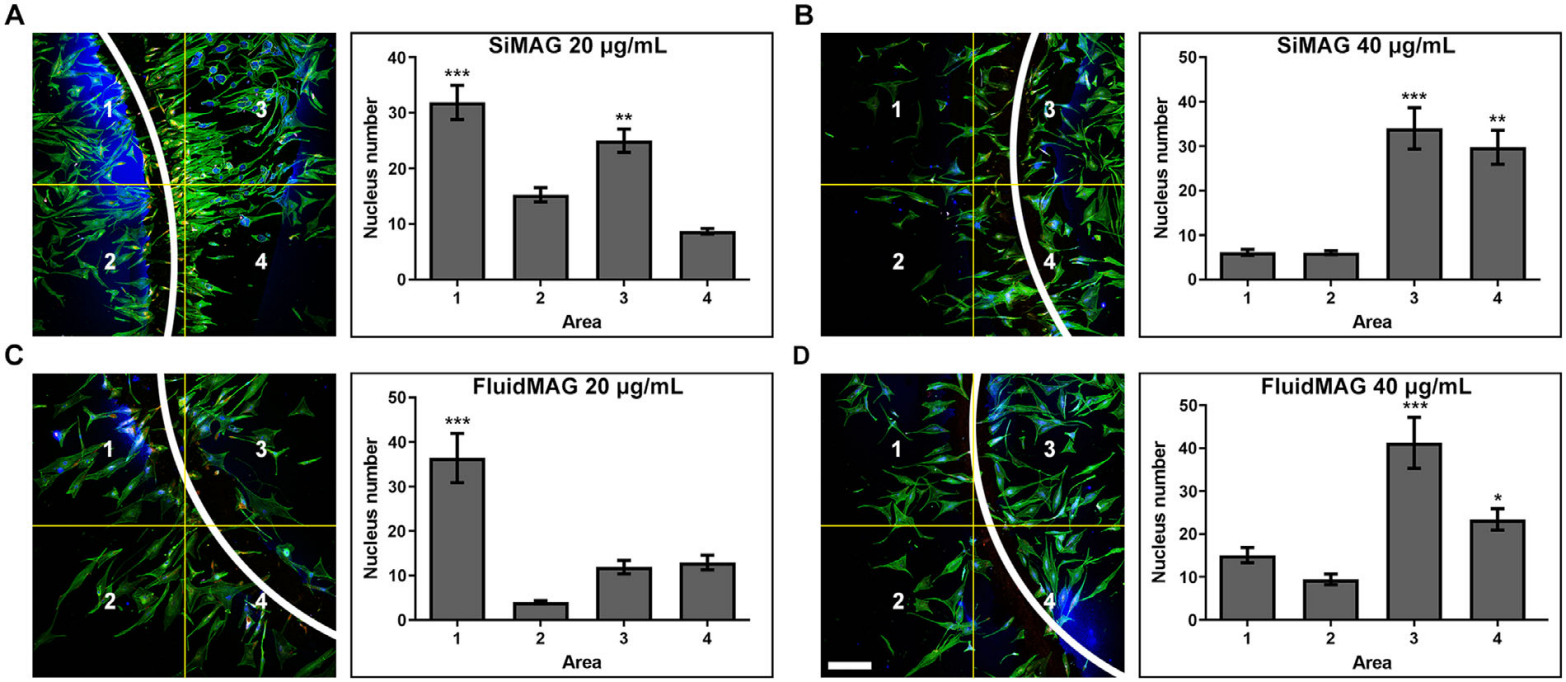
Control of cell growth density by application of magnetic particles. Magnetic particle-loaded primary cultured HCECs were suspended and cultured in the plate under which a round neodymium magnet was placed. Loading HCECs with 20 or 40 µg/mL of SiMAG or fluidMAG efficiently increased cell population near the magnet area. (**A** and **B**) HCECs loaded with 20 or 40 µg/mL of SiMAG. An increased cell population was observed in areas near the border of magnet. (**C** and **D**) HCECs loaded with 20 or 40 µg/mL of fluidMAG. An increase of cell population was observed in areas near the border of magnet. The white arc in the panel indicates the margin of the round neodymium magnet. HCECs in (**A**–**D**) were stained with F-actin (green) and DAPI (blue). *P* values were calculated by comparing to lowest populated area: area 4 in (**A**) and area 2 in (**B**, **C**, and **D**). Triplicates of each treatment group were performed. Representative figures are presented. *P < 0.05, **P < 0.01, ***P < 0.001.

The application of 20 µg/mL of SiMAG or fluidMAG was sufficient to effectively attract immortalized HCECs (B4G24 cells) near the magnet area (Fig. 7). Near the magnet area, cell density was significantly increased (Supplementary Fig. S1). In SiMAG applied cells, cell densities (cells/quadrant) measured at the most populated area and the least populated area were 29.0 and 3.6, respectively (*P* < 0.001). In fluidMAG applied cells, cell densities measured at the most populated area and the least populated area were 26.8 and 2.4, respectively (*P* < 0.001). Both 20 µg/mL and 40 µg/mL of SiMAG or 40 µg/mL of fluidMAG were effective in controlling the migration of primary culture of HCEC to the magnet area (Fig. 8). In SiMAG (40 µg/mL) applied cells, cell densities measured at the most populated area and the least populated area were 34.0 and 6.1, respectively (*P* < 0.001). In fluidMAG (40 µg/mL) applied cells, cell densities measured at the most populated area and the least populated area were 41.2 and 9.7, respectively (*P* < 0.001). These findings suggest that HCECs with magnetic particles can gather near the magnet area according to the magnetic field and settle in the culture plate.

## Discussion

In this study, we investigated the short-term effects of magnetic microparticles on HCECs using an established cell line and primary cultured cells. We found that exposure to SiMAG or fluidMAG at concentrations of up to 20 µg/mL for 48 hours did not affect the viabilities of immortalized or primary culture of HCECs. These cells could relatively tolerate exposure to SiMAG and fluidMAG at concentrations of up to 10 µg/mL for 72 hours, with a maximum loss of viability of 25%. Cellular tight junction ZO-1 expression remained intact in HCECs after exposure to magnetic particles at concentrations up to 20 µg/mL. In addition, an ex vivo study revealed that cells could tolerate exposure to 20 µg/mL of SiMAG or fluidMAG for 72 hours, showing no patchy loss of HCECs. Taken together, these results indicate that the safe concentration of SiMAG and fluidMAG for HCECs is up to 20 µg/mL when they are applied for 48 hours. This means that these particles within a safe range could be used within 48 hours in HCEC injection therapy or tissue engineered HCEC constructs. The application of magnetic microparticles combined with a magnetic field successfully increased cell migration near the neodymium magnet, suggesting that an increase of HCEC density may be possible.

Magnetic particles, although not yet at the clinical stage, can be applied to HCECs in the setting of cell injection therapy or fabrication of bioengineered HCECs cell sheet for the treatment of corneal endothelial cell failure in the future.^12^^,^^21^^,^^22^ Unlike penetrating keratoplasty or endothelial keratoplasty, in which unmodified corneal endothelial cells are directly transplanted to the recipient cornea, these methods can amplify HCECs in the laboratory and apply various cell engineering techniques to enhance HCEC function or adhesion.^23^^,^^24^ Because of the potential advantages, various previous studies have attempted to control the movement of cornea endothelial cells using magnetic particles.^9^^,^^10^^,^^14^^,^^15^^,^^25^

The effect of magnetic nanoparticles on HCECs culture was previously investigated by Moysidis et al.^10^ They used 50-nm-sized magnetic nanoparticles (rat anti-mouse IgG1 superparamagnetic MACS MicroBeads, 50 nm diameter; Miltenyi Biotec, Inc., Bergisch Gladbach, Germany) and found that magnetic nanoparticle-loaded HCECs could be cultured more densely by 2.4-fold compared with the control HCECs. In addition, the magnetic particle-loaded HCECs formed a uniform monolayer with intact expression of the ZO-1 protein. Furthermore, they found that the movement speed of HCECs under the magnetic field increased proportionally with the concentration of magnetic particles. Although they reported no significant changes of HCECs viability after exposure to magnetic nanoparticles, exact concentrations of Microbeads were unknown as they were sold in the size of enough antibody number to isolate 1 × 10^9^ total cells using one vial of product. Additionally, they observed cell viability only after 24 hours of exposure to magnetic particles, which made direct comparison of their viability data with our SiMAG/fluidMAG data impossible.

Cornell et al.^25^ have used dextran superparamagnetic iron oxide nanoparticles (50 nm sized, an iron core of 7–10 nanometers coated with biotin; Micromod, Rostock, Germany) and labeled bovine cornea endothelial cells. Then, they applied a neodymium magnet for 3 days to superparamagnetic iron oxide nanoparticle-labeled cornea endothelial cells and observed that the cell viability was well-maintained, even after magnet exposure with an intact cytoskeleton. However, they found that cell viability decreased by approximately 10% when cells were cultured with 100 × 10^6^ nanoparticles per cell. Differences between animal-derived cells and human-derived cells made direct comparison with our current results difficult.

The effect of magnetic particle on HCEC injection therapy have been studied by Mimura et al.^15^ and Xia et al.^9^ in rabbits, respectively. Mimura et al.^15^ labeled rabbit cornea endothelial cells with spherical iron powder purchased from Cosmo bio Inc. (Tokyo, Japan) (size information is unavailable). Iron powder-loaded cells were then injected into the anterior chamber of the rabbit. A neodymium magnet was then attached at the eyelid to enhance cell attachment on the posterior corneal surface. They observed that iron powder-laden cornea endothelial cells attached better to the posterior cornea surface and successfully restored corneal transparency. However, high concentrations of iron powder were found to be toxic. They showed the viability of rabbit corneal endothelial cells decreased more than 50% when spherical iron powder concentration exceeded 10 µM. Xia et al.^9^ injected superparamagnetic nanoparticle-loaded (rat anti-mouse IgG1 superparamagnetic nanoparticles) HCECs into the anterior chamber of a rabbit with endothelial cell decompensation and applied a neodymium magnet outside the closed eyelid for 3 hours. They observed successful HCEC lining of the posterior surface of rabbit cornea with restoration of corneal transparency. However, safety issues, such as immune reactions against animal-derived proteins, were not resolved completely in previous studies.

Possible toxicity of magnetic micro- and nanoparticles to cornea endothelial cells was investigated by Raju et al.^14^ after injecting particles into the anterior chambers of rats. They used 50-nm-sized nanoparticles (55%–59% iron oxide w/v) that were superparamagnetic, dextran-coated beads conjugated to goat anti-mouse IgG (Miltenyi Biotec Inc.). In their investigation, 4-µm-sized magnetic microparticles (Dynabeads M-450, tosyl-activated, 37% iron oxide w/v; Invitrogen, Dynal, Norway) were also included. Although they found no significant toxicity of 50-nm-sized magnetic nanoparticles to cornea endothelial cells, the injection of 4-µm-sized magnetic microparticles decreased cornea endothelial cell density significantly after 1 week and 5 months, which raised the concern of possible toxic effect in a long-term observation.

To use nanoparticles in the clinical field, the most important thing is safety. The smaller the size of nanoparticles, the easier it is to freely invade the organelle inside the cell. Therefore, special attention should be given to the size of nanoparticles. Furthermore, cellular uptake of magnetic nanoparticles depends on the cell type and surface properties of nanoparticles.^26^ Of note, HCECs are different from animal corneal endothelial cells in that HCECs in vivo lack a proliferative capacity.^2^^,^^27^ The nature of magnetic particles is also important. Previous studies by Moysidis et al.^10^ and Xia et al.^9^ have used animal-derived immunoglobulin as magnetic particles. Animal proteins, such as immunoglobulins used in several previous studies,^9^^,^^10^^,^^14^ have the potential to induce immune reaction when they are used in humans. Uveitis caused by immune reaction is an important risk factor of corneal endothelial transplantation failure.^28^ Therefore, ideal magnetic nano- or microparticles for HCECs should be free of immunogenicity.

We used SiMAG and fluidMAG in this study. These magnetic nano- and microparticles are commercially available and pure inorganic compounds. These particles have been actively investigated in in vivo magnetic resonance imaging, biomagnetic separation of cells, and cell targeting therapy.^29^^–^^33^ Their safety and efficacy data have been somewhat accumulated through various animal model, organ culture, and cell culture. The safety and efficacy of magnetic particles have been previously verified to be free of immunogenicity in the retina.^34^ However, the application of these particles to HCECs requires a new set of verification experiments. To the authors’ knowledge, this is the first time that these particles are applied to HCECs and that the control of HCEC movement is verified. It is very encouraging to find that these particles are safe to primary cultured HCECs and ex vivo human corneas for up to 72 hours in a range of concentrations. Furthermore, active control of HCEC movement was verified in cell culture with the application of a neodymium magnet.

In this study, various settings, such as immortalized HCECs, primary cultured HCECs, and ex vivo human corneas, were used to evaluate effects of SiMAG and fluildMAG. Slight differences in the safety range of SiMAG and fluildMAG among different settings might be caused by changes induced in the immortalization process of B4G12 cells, single cell separation and subsequent passages of culture, and senescence change during passages of primary culture HCECs.^35^^,^^36^ In the case of primary culture HCECs, the amount was insufficient to perform all culture experiments in the current study, especially protein analysis, because senescence accelerated as passages of culture continued.^37^ Of course, it was not easy to obtain a good number of research human corneas in good condition for cell culture.

The significance of our study was that it demonstrated short-term effects of magnetic particles not verified yet in the field of ophthalmology using human-derived corneal endothelial cells. However, if we added another negative control, such as HCECs with magnetic particle treatment without magnet application or HCECs with magnet application without magnetic particles treatment, we would have further verified cell migration toward the magnet. In addition, the inability to confirm safety and efficacy in vivo through animal experiments is a clear limitation of this study. An in-depth study on whether intracellular magnetic particles are maintained or continuously decreased over several months or years and whether there is any damage to delicate intraocular tissues by magnetic particles released from cells is essential. Human-derived endothelial cell transplantation in animal models can induce inadvertent immune rejection.^38^^,^^39^ Therefore, an accurate assessment of safety and efficacy might be impossible. Another drawback of this study was that it did not have specific data on how far and at what speed it was possible to control the movement of cells.

In conclusion, we verified short-term effects of SiMAG and fluidMAG in HCECS and their availability to control the movement of HCECs with an external magnetic field. It is important to accumulate data on the safety and effectiveness of new magnetic particles for ophthalmic use. Considering recent vigorous attempts to treat corneal endothelial cell insufficiency using cellular bioengineering, our findings will be helpful for future development of novel bioengineering techniques for corneal endothelial cell culture or cell injection therapy using magnetic particles.

## Supplementary Material

Supplement 1

Supplement 2
