# Supplementary material for: Effect of Magnetic Microparticles on Cultivated Human Corneal Endothelial Cells
Source: Transl Vis Sci Technol. 2023 Feb 9;12(2):14. doi: 10.1167/tvst.12.2.14 (PMC9924430; doi:10.1167/tvst.12.2.14)

Supplementary Figure 1. Phase contrast microscopic image of cultured HCECs after application of SiMAG or fluidMAG under the influence of magnet. Brown pigments inside cells are magnetic particles (SiMAG or fluidMAG). These pictures show that cells with brown pigments are dragged to the magnet area.

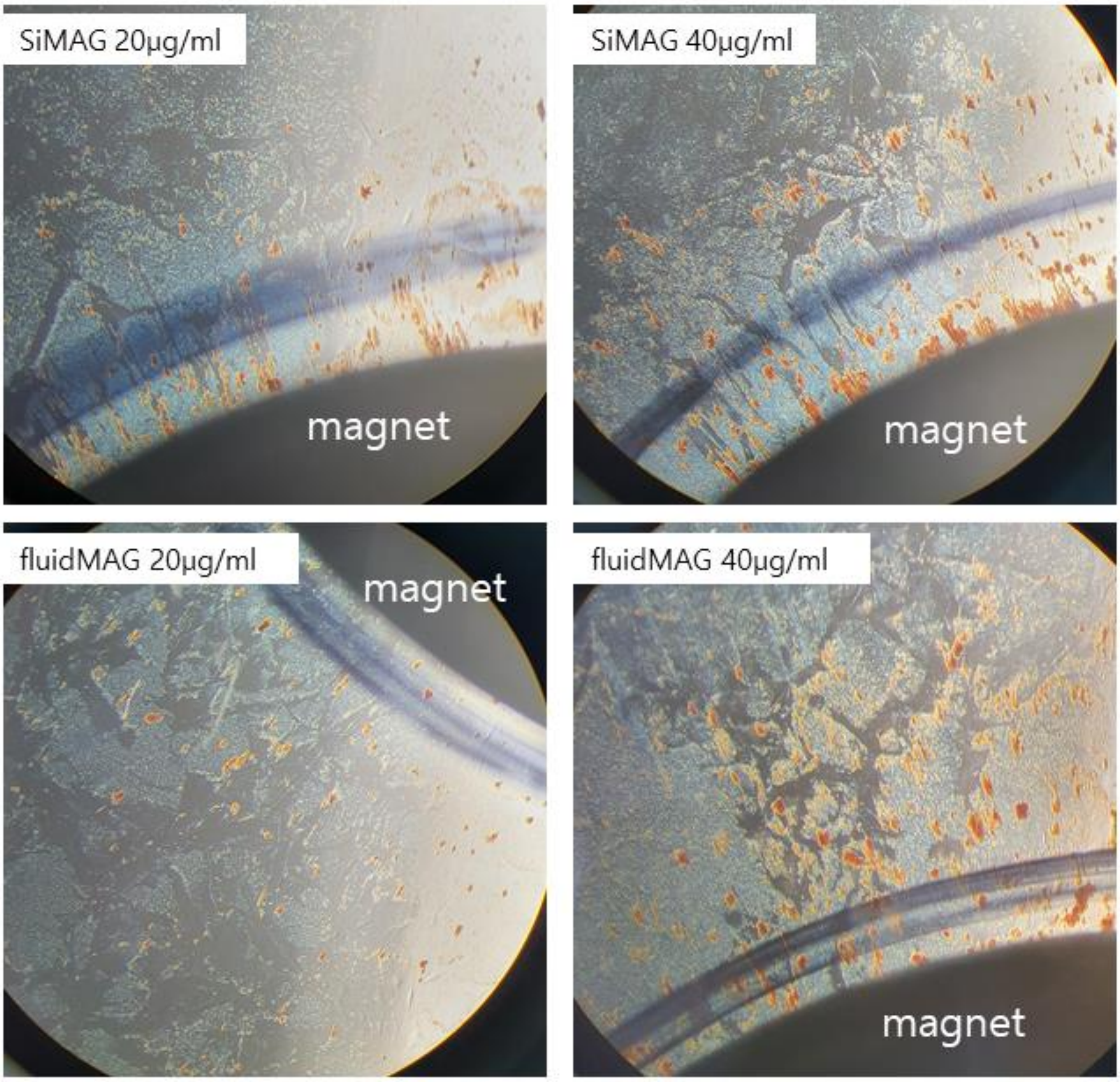

Supplement: Supplement 1 [file tvst-12-2-14_s001.pdf]
