# Supplementary material for: Effect of Magnetic Microparticles on Cultivated Human Corneal Endothelial Cells
Source: Transl Vis Sci Technol. 2023 Feb 9;12(2):14. doi: 10.1167/tvst.12.2.14 (PMC9924430; doi:10.1167/tvst.12.2.14)

Supplementary Figure 2. A: Plates with cells loaded with magnetic particles without applying a magnet. Cells are relatively evenly distributed in the center of the chamber. **B and C**: A magnet was applied to SiMAG (B) and fluidMAG (C) containing cells before cells attached on the plate. After removing the magnet, it was found that floating SiMAG or fluidMAG were densely gathered around the magnet area. Therefore, there was no space for the cell to attach on the plate near the magnet.

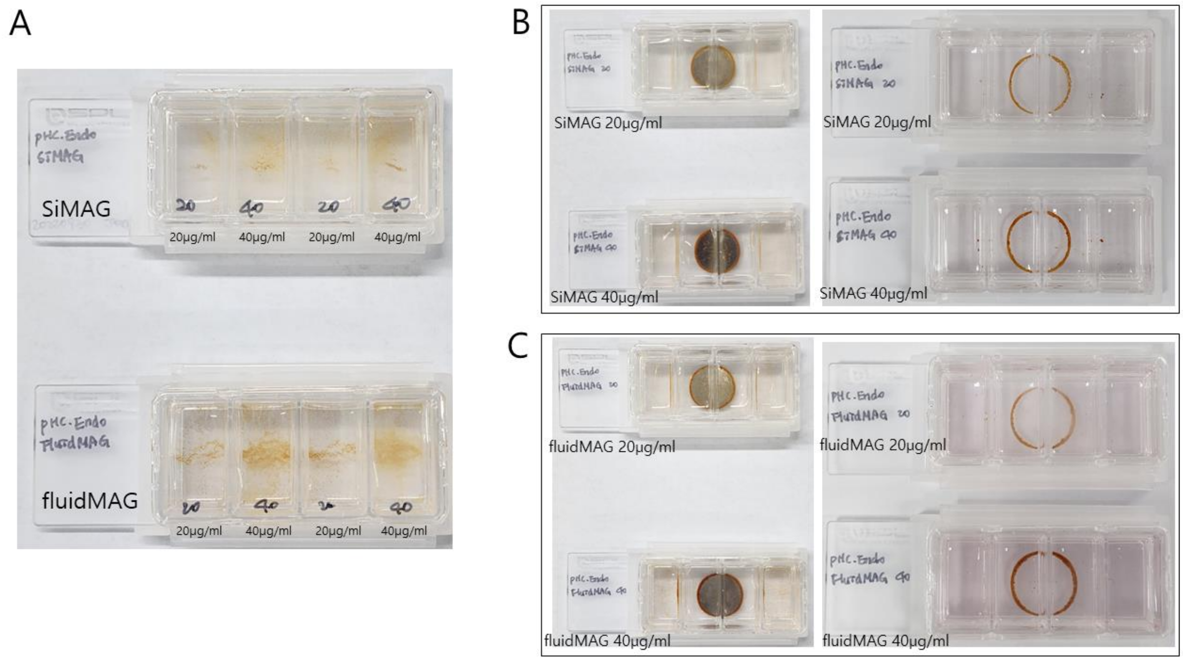

Supplement: Supplement 2 [file tvst-12-2-14_s002.pdf]
